# Differential DNA methylation patterns in whole blood from ACPA-positive patients with DMARD-naïve rheumatoid arthritis at clinical disease onset

**DOI:** 10.3389/fimmu.2025.1488161

**Published:** 2025-07-21

**Authors:** Anders Jørgen Svendsen, Jonas Mengel-From, Peter Junker, Christine Dalgård, George Davey Smith, Caroline L. Relton, Hannah R. Elliott, Kirsten Kyvik, Hanne Lindegaard, Anne Friesgaard Christensen, Qihua Tan

**Affiliations:** ^1^ Research Unit for Epidemiology, Biostatistics and Biodemography, Department of Public Health, University of Southern Denmark, Odense, Denmark; ^2^ Department of Internal Medicine & Emergency, Odense University Hospital, Svendborg, Denmark; ^3^ Unit of Human Genetics, Department of Clinical Research, University of Southern Denmark, Odense, Denmark; ^4^ Department of Rheumatology, Odense University Hospital, Odense, Denmark; ^5^ Clinical Pharmacology, Pharmacy and Environmental Medicine, Department of Public Health, University of Southern Denmark, Odense, Denmark; ^6^ MRC Integrative Epidemiology Unit, Bristol Medical School, University of Bristol, Bristol, United Kingdom; ^7^ Population Health Sciences, Bristol Medical School, University of Bristol, Bristol, United Kingdom; ^8^ Research Unit of Clinical Genetics, University of Southern Denmark, Odense, Denmark; ^9^ Department of Internal Medicine, Lillebaelt Hospital, Kolding, Denmark

**Keywords:** rheumatoid arthritis, anti-CCP antibodies, epigenetics, DNA-methylation, incidence

## Abstract

**Objective:**

Epigenetic DNA imprints are increasingly being recognized as co-drivers of disease in complex conditions. In this exploratory and hypothesis-generating epigenome-wide association study (EWAS), we investigated differential methylation patterns in peripheral blood leucocytes from patients with early untreated ACPA-positive rheumatoid arthritis (RA) versus controls.

**Methods:**

Whole blood DNA was isolated from 101 disease-modifying anti-rheumatic drug (DMARD)-naïve patients with recent clinical onset of ACPA-positive RA and 200 controls. DNA methylation was studied using the Illumina MethylationEPIC BeadChips (Illumina). We assessed our findings against previously reported differentially methylated DNA positions associated with RA including an EWAS on peripheral blood leucocytes from a similar Drop Nordic cohort.

**Results:**

We identified 16,583 CpG sites and 14 differentially methylated regions (DMRs) associated with RA. The most robust DMRs were in the gene body of *LAMP1* and the *TNSF14* GENE known as *LIGHT*. We identified three novel Kyoto Encyclopedia of Genes and Genomes (KEGG) pathways, the taste transduction pathway, the olfactory pathway, and the viral carcinogenesis pathway, which have not previously been associated with RA. We replicated 2,248 CpG sites reported earlier in an EWAS on peripheral blood leukocytes from RA patients of Scandinavian ancestry with incipient untreated ACPA-positive disease.

**Conclusion:**

We have detected a considerable number of epigenetic marks with potential relevance to the pathogenesis of RA. These findings may pave the way for the development of narrowly targeted new drugs and possibly assist to retrieve persons at particular risk of acquiring RA.

## 1 Introduction

Rheumatoid arthritis (RA) is a chronic, systemic immune-mediated disease of unknown etiology. Symmetrical small joint synovitis in hands and feet is a key clinical presentation and a core item in internationally endorsed classification criteria. Notably, asymptomatic immune dysfunction reflected as, e.g., occurrence of autoantibodies, like rheumatoid, rheumatoid factor (IgM-RF) and antibodies against citrullinated proteins (ACPA), often precedes clinical onset by several years. Moreover, RA is a dynamic disease that is capable of spreading to previously uninvolved synovial joints and to extra-articular sites, often accompanied by the clinically silent emergence of comorbid conditions like premature arteriosclerosis (1). In recent years, evidence has accumulated indicating that RA should be conceived as a disease entity comprising separate subsets that despite shared clinical features are characterized by distinctive pathogenetic mechanisms and clinical presentations, particularly regarding the presence or absence of autoantibodies commonly referred to as seropositive and seronegative RA. Joint destruction and comorbidities such as cardiovascular disease and extra-articular manifestations are most prominent in the seropositive subset of patients (60% to 80% of RA cases) (2). The strongest genetic risk factor for RA identified so far is the shared epitope, a five-amino-acid sequence motif encoded by RA-associated alleles in the human leukocyte antigen complex (3). This association is primarily restricted to ACPA-positive RA. The strongest environmental risk factor for RA is smoking, which is also mainly associated with ACPA-positive RA. There is a strong interaction effect between these two risk factors. Thus, patients carrying the shared epitope and who have ever been exposed to smoking have an increased risk of ACPA-positive RA by 20-fold or more compared with non-smokers who do not carry the shared epitope (4).

Heritability estimates on RA based on twin studies have yielded considerably varying results ranging between 12% and 60% (5) while a genome-wide association study (GWAS) estimated the heritability at 52% (6). There is an overall agreement that the known alleles and polygenic signals account for 35% of the total liability to acquire RA, which falls short of heritability estimates of approximately 50%. This so-called missing heritability does not necessarily reflect absence of genetic variants, because current estimates of heritability may be inflated by disregarding, e.g., both gene–gene and gene–environment interaction (7). Currently, there is no substantial evidence to assume the existence of transgenerational epigenetic inheritance (8), but more research is needed to explore this issue.

The rather low RA concordance rate in monozygotic twins has fueled interest in the study of epigenetic DNA marks of potential environmental origin as risk factors and disease modifiers in RA development. Thus, we have previously reported that methylation patterns differ between monozygotic twins with and without RA (9).

In recent years, there has been growing interest in the role of epigenetic mechanisms in RA development. Such DNA modifications may serve as dynamic links between genotype, environment, and phenotype. In humans, DNA methylation has been studied most extensively, and so the best-known function of DNA methylation is to change cis regulatory elements, usually located upstream of genes. Several epigenome-wide association studies (EWASs) have identified differentially methylated loci and regions in RA, and candidate gene methylation changes have been observed in genes involved in immune regulation, cytokine signaling, and cell adhesion (10).

EWASs investigate the association between a phenotype and DNA epigenetic changes scattered across the whole genome (11). Several EWASs on RA have been undertaken but often with cases from different disease subsets, e.g., incident cases versus prevalent cases, and different materials have been used, e.g., whole blood versus PBMCs versus cell-sorted samples and synovial fibroblasts. Environmental diversity between study participants may also contribute and immune-modulating therapies may have impacted the epigenetic patterns.

In this exploratory case–control study, with a reasonable sample size and an optimized case:control ratio, we aimed to investigate DNA methylation patterns in peripheral blood cells from patients with newly diagnosed ACPA-positive and treatment-naïve [disease-modifying anti-rheumatic drugs (DMARDs) and glucocorticoids] RA patients versus controls.

## 2 Materials and methods

### 2.1 Cases and controls

The study comprised 101 patients with newly diagnosed ACPA-positive RA with symptom duration less than 1 year and all treatment naive (**Table 1**) and fulfilling the ACR (American College of Rheumatology) 1987 revised criteria for RA (12). Blood samples were collected at the time of diagnosis when all the patients had active disease and therefore swollen joints. The study was conducted in accordance with the Declaration of Helsinki, and local ethics committee approval was obtained in advance. All the patients and controls were of Scandinavian genetic ancestry and were included from two outpatient clinics in the Region of Southern Denmark. Patients with current infection, malignancies apart from non-melanoma skin cancer, autoimmune diseases, and recent surgery were excluded (13–15).

**Table 1:** Descriptive statistics of samples.

	Case	Control	*p*-value*
Sample size	101	213	
Sex			
Male	35	101	0.03
Female	66	112	
Age			
Min	20	19	<0.01
Max	76	65	
Mean	52	37	
Smoking			
Yes	69	87	<0.01
No	31	119	
Missing	1	7	

*p-value based on logistic regression.

A total of 200 control individuals were recruited from the GEMINAKAR study and consisted of a random selection of self-reportedly healthy monozygotic twin pairs born in 1931–1982 (16). Thus, control twins with, e.g., autoimmune diseases including RA were excluded. Genomic DNA was used from only one twin individual who was chosen at random from each pair.

The RA and control samples were shuffled in the lab experiment. The 200 controls used in this study were random unrelated singletons of a large healthy cohort of DNA methylation data on 958 twin samples. The mixture of RA samples and 958 healthy samples during lab work and the random sampling of the 200 controls from the 958 healthy samples after DNA methylation analysis in this study avoid specific batch effects.

### 2.2 Blood sample collection

Genomic DNA was isolated from EDTA-treated whole peripheral blood and kept at −80°C until use. DNA purification was done by either a standard salting out method (17) or the Promegas Maxwell 16 method. We have not been able to find evidence in the literature that the DNA purification methods used have any impact on methylation arrays and Illumina has, on request, informed us that any standard DNA extraction method or kit that provides high-quality genomic DNA is suitable for Infinium Arrays. Moreover, we made multi-dimensional scaling on the data and did not find evidence of specific batch effect in the data used (**Supplementary Figure S1**).

### 2.3 DNA methylation analysis using Infinium methylation EPIC v1.0

2.3.1

This information is described in **Supplementary File 1**.

### 2.4 Statistical analysis

#### 2.4.1 Single-CpG-based analysis

We applied the linear mixed-effect model to study the association between DNA methylation (DNAm) (dependent variable) and RA. We also included smoking and its interaction with RA to investigate smoking-dependent associations between DNAm and RA. The model was adjusted for age, sex, and cell composition (cells) by including them as model covariates. The random-effect variables were defined for batches (plate and well) and sample location on the array. Smoking (SMK) was defined as ever smoker versus never. Age was defined as age at blood sampling. To control for multiple testing, we calculated the false discovery rate (FDR) using the R function “p. adjusts”. We defined *p* < 1e−05 as suggestive significance and FDR < 0.05 as genome-wide significance. Only results that fulfilled these statistical criteria are considered in the manuscript except for pathway analysis, which relies on the gene set enrichment analysis (GSEA) (18).

#### 2.4.2 Multiple-CpG-based analysis

In addition to the single-CpG-based analysis, we extended our analysis to multiple CpGs in order to search for differentially methylated genomic regions (DMRs) associated with RA. This was done using the bumphunter approach introduced by Jaffe et al. (19) as included in the R package minfi. This approach assumes that the locus-specific estimates of regression coefficients (βs) are smooth along the strand of a chromosome and applies the loess smoothing technique to smooth coefficient βs within a pre-defined chromosomal region (300 base pairs in our analysis). After smoothing, the 99th percentile of the smoothed βs can be calculated to obtain upper and lower thresholds. These thresholds are then used to define hyper- or hypomethylated DMRs with smoothed peaks above or below the thresholds. For each DMR identified, a sum statistic was calculated by summing the absolute values of all the smoothed βs within the region being studied. The sum statistic was subsequently used to rank all DMRs with the DMR of the highest sum statistic value as the most important. Statistical significance of the DMRs was assessed by computer permutation (we set 1,000 replications) in combination with correction for multiple testing to obtain family-wise error rate etc. (FWER) (19).

#### 2.4.3 Hypergeometric test

We applied the hypergeometric test for over-representation analysis (ORA) to assess if the overlaps of identified markers with markers reported from previous studies are significantly different from our EWAS results. The R function “phyper” was used for calculating the hypergeometric probability.

### 2.5 Functional annotation

We performed GSEA (18) to test if specific functional clusters or gene sets are enriched by the EWAS estimates. Following the steps in GSEA, we estimated an enrichment score for each gene set and then calculated its statistical significance using a permutation test with 1,000 random replicates. The R package clusterProfiler (20) was used for GSEA on Gene Ontology (GO) terms and on biological pathways from the Kyoto Encyclopedia of Genes and Genomes (KEGG), respectively. Likewise, the significance of functional clusters was defined following correction for multiple testing using FDR < 0.05.

In addition to GSEA, we also applied the pathway analysis method proposed by Phipson et al. (21), which takes into account the varying numbers of CpG probes in different genes and which calculates the hypergeometric probability by ORA of genes in a particular pathway using the missMethyl package in R.

### 2.6 Analysis of global and locational trend of DNA methylation

Based on EWAS results of single CpG sites, we additionally analyzed the overall trend of hyper or hypo-methylation by testing the enrichment of significantly differentially methylated CpGs in the overall rank of test statistics of all CpGs in the EWAS. We tested the general trend by each location of CpGs divided as promoter (TSS200, TSS1500, 1stExon, and 5’UTR), gene body (body, 3’UTR, and ExonBnd, i.e., exon boundaries), intergenic regions, and for all locations combined (global). The function geneSetTest () implemented in the R package limm was used for the test.

## 3 Results

### 3.1 Single-site-based EWAS

**Figure 1:**
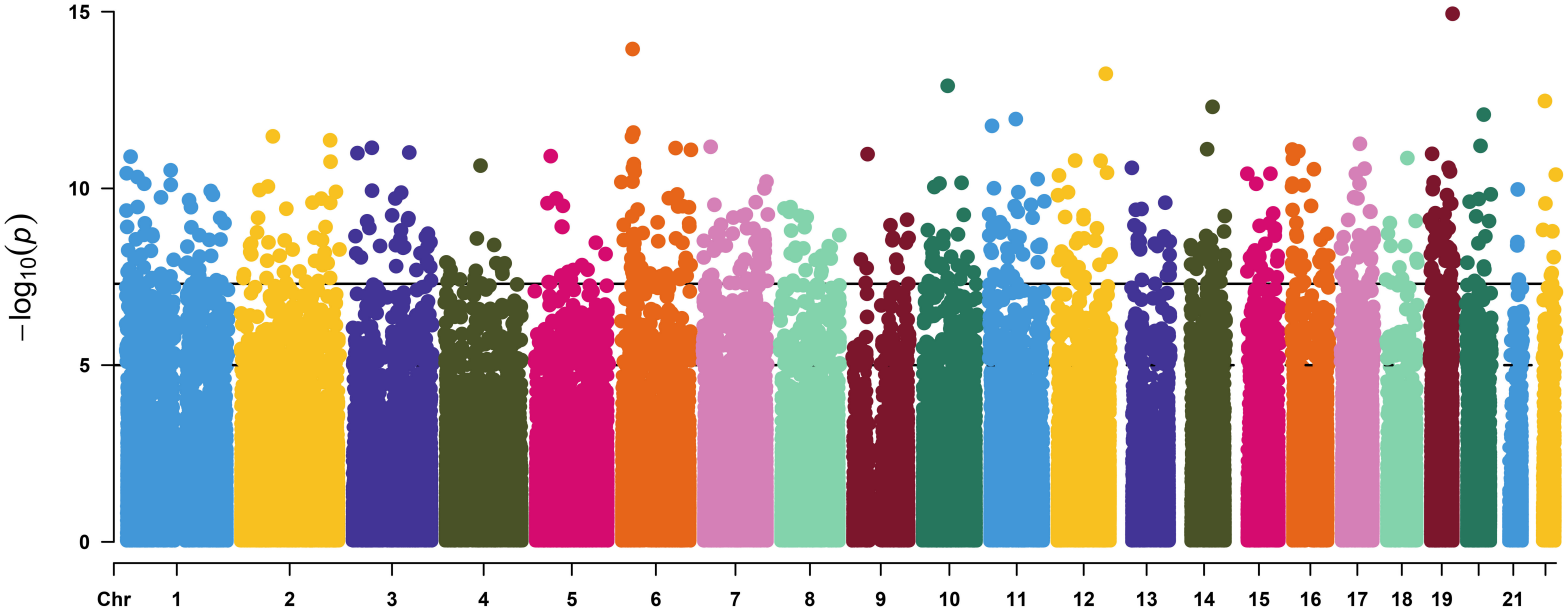
Manhattan plot of the linear mixed model of DNA methylation and rheumatoid arthritis. The vertical axis represents the –log10(P value of the mixed effect model) versus genomic position for RA-associated CpGs. The solid horizontal line represents genome-wide significance of unadjusted p value = 5.0 × 10 −8 (612 sites) and the dash-dotted line represent suggestive significance of unadjusted p value = 1.0 × 10 −5.

**Figure 2:**
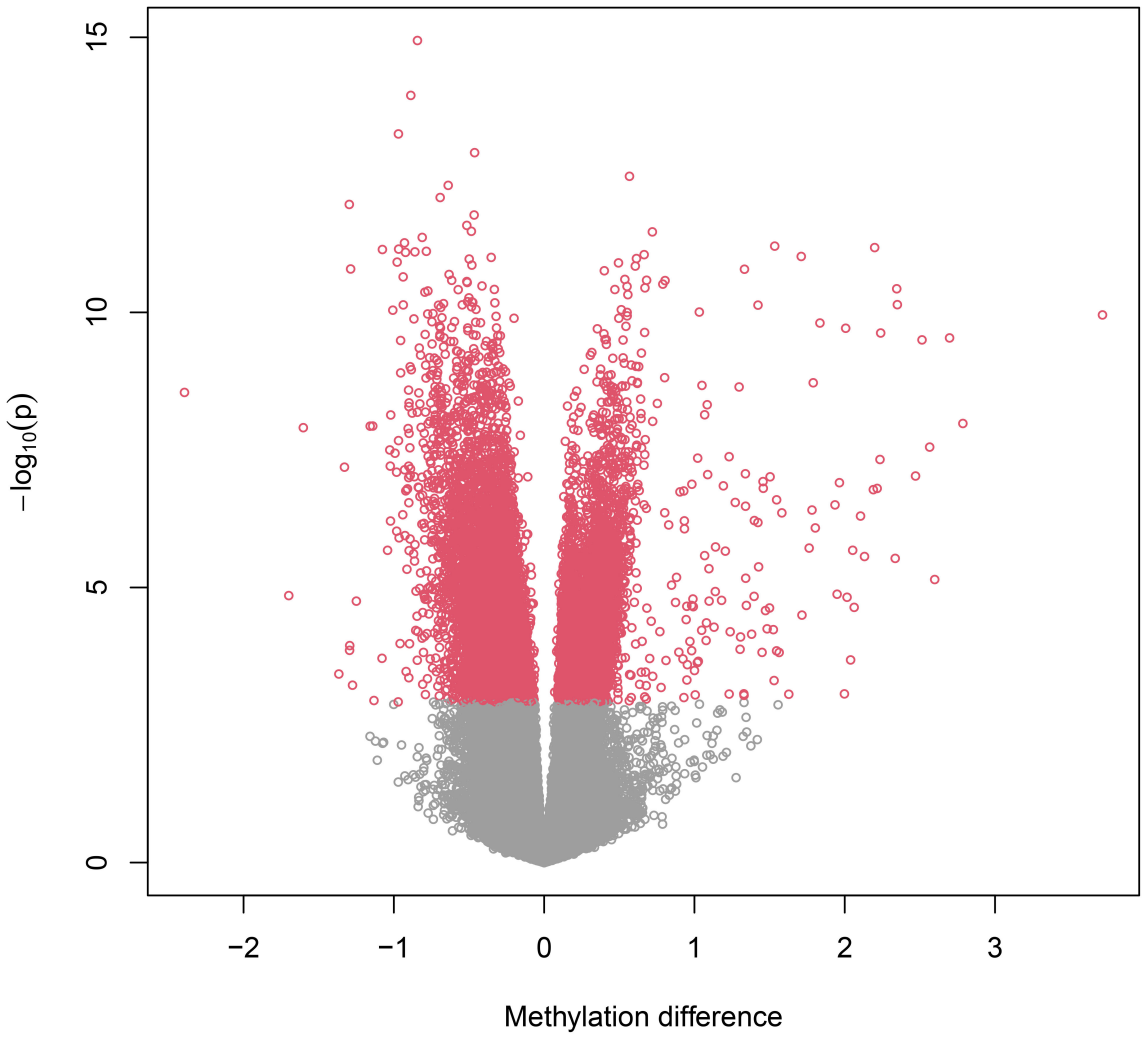
Volcano plot. Volcano plot representing differentially methylated probes. The negative dots on the horizontal axis represent hypomethylated probes in RA and positive dots hypermethylated probes in RA. The vertical axis represents the -log10 p value. The figure indicates a major methylation deficiency in the RA methylome.

By applying the linear mixed model to the DNA methylation data, we identified a total of 16,583 CpG (16k/800k = 2%) sites that were differentially methylated for RA with genome-wide significance of FDR<0.05 (**Supplementary Table S1**). Among these sites, 612 CpGs came out as highly significant (unadjusted *p* < 5e−08), as illustrated by the enlarged dots in the Manhattan plot (**Figure 1**). As shown with red dots in the volcano plot (**Figure 2**), there is an excess of hypomethylated CpGs in RA patients indicating a methylation deficiency in the RA DNA methylome. Therefore, as a next step, we wanted to explore the distribution of excess hypomethylation according to promotor region, gene body, and intergenic region using the geneSetTest() function in limma. CpGs annotated to multiple gene regions were excluded from this analysis (**Supplementary Table S2**). Thus, 62% of the CpGs were hypomethylated in the promotor region, 54.6% in the gene bodies, and 57.3% in the intergenic regions. For all three regions, the deviation from the frequency expected by change was strong.

The top 70 strongest differentially methylated CpGs are presented in **Table 2**. Interestingly, we also identified multiple CpGs that were differentially methylated for smoking with the highest significance at *p* < 3.85e−20 for site cg05575921 located in the gene body of AHRR. A total of 21 additional CpGs reached genome-wide significance with FDR < 0.05 (**Supplementary Table S3**). Of particular note, the CpGs associated with smoking also displayed a markedly reduced DNA methylation pattern. While cg05575921 was previously identified to be hypomethylated among smoking individuals (22), no statistically significant interaction was identified between RA and smoking (*p*-value = 0.562).

**Table 2:** Top 70 most statistically significant CpG sites differentially methylated for RA.

Probe ID	Coeff	SE	*t*-value	*p*-value	Chromosome number	Position	Relation to island	UCSC ref gene name	UCSC ref gene group
cg15162827	−0.84	0.10	−8.68	1.14E−15	19	55897108	Island	RPL28	TSS200
cg04796146	−0.89	0.11	−8.24	1.14E−14	6	28831735	Island		
cg27553372	−0.97	0.12	−7.94	5.71E−14	12	120731091	OpenSea		
cg13565382	−0.46	0.06	−8.15	1.25E−13	10	63628787	OpenSea		
cg16132339	0.57	0.07	7.63	3.36E−13	22	24313637	N_Shelf	DDTL	3’UTR
cg01511804	−0.64	0.08	−7.65	4.92E−13	14	74318438	Island	PTGR2	TSS200
cg08548498	−0.69	0.09	−7.63	8.17E−13	20	43883546	OpenSea	SLPI	TSS1500
cg04378603	−1.30	0.16	−8.06	1.09E−12	11	65266494	S_Shore	MALAT1	Body
cg08185661	−0.47	0.06	−7.41	1.70E−12	11	7273498	Island	SYT9	1stExon
cg00985729	−0.51	0.07	−7.40	2.62E−12	6	30712559	S_Shore	IER3	TSS1500
cg25636665	−0.48	0.07	−7.32	3.36E−12	2	80549579	Island	CTNNA2	Body
cg13434361	0.72	0.10	7.33	3.44E−12	6	27197402	OpenSea		
cg13315450	−0.81	0.11	−7.48	4.34E−12	2	220114289	N_Shelf	STK16	Body;3’UTR
cg09000583	−0.93	0.13	−7.19	5.45E−12	17	46802888	Island	HOXB13	3’UTR
cg03191045	1.53	0.21	7.17	6.24E−12	20	36040903	OpenSea		
cg26818735	2.20	0.30	7.22	6.64E−12	7	19156621	Island	TWIST1	1stExon
cg14986395	−0.97	0.14	−7.16	7.08E−12	3	49824225	Island	IP6K1	TSS1500
cg14817541	−1.08	0.15	−7.17	7.20E−12	6	133563868	Island	EYA4	5’UTR
cg18247042	−0.78	0.11	−7.26	7.76E−12	14	61116506	Island	SIX1	TSS1500
cg08977639	−0.86	0.12	−7.41	7.94E−12	16	1047820	Island		
cg25135198	−0.92	0.13	−7.32	8.11E−12	6	170862522	N_Shore	PSMB1; TBP	TSS200; TSS1500
cg09559352	0.67	0.09	7.12	8.93E−12	16	15978691	N_Shelf	C16orf63	Body
cg24295125	1.71	0.24	7.10	9.63E−12	3	140950234	Island	ACPL2	TSS1500
cg26770187	−0.35	0.05	−7.17	1.00E−11	3	14693171	Island	C3orf19	TSS200
cg21195395	0.61	0.09	7.17	1.05E−11	19	5981162	S_Shelf		
cg04804550	−0.50	0.07	−7.20	1.07E−11	9	38424609	S_Shore	IGFBPL1	TSS200
cg23455224	−0.98	0.13	−7.26	1.22E−11	5	39075128	Island	RICTOR	TSS1500
cg06967016	0.49	0.07	7.05	1.26E−11	1	12039049	N_Shore	MFN2	TSS1500
cg06958567	−0.48	0.07	−7.09	1.38E−11	18	52495541	N_Shore	RAB27B	TSS1500
cg06841964	0.61	0.09	7.06	1.44E−11	16	2635445	S_Shore	PDPK1	Body
cg23465749	−1.29	0.18	−7.25	1.63E−11	12	46123553	Island	ARID2	TSS200
cg18532726	1.33	0.19	7.11	1.64E−11	12	108079562	Island	PWP1	TSS200
cg20211362	0.40	0.06	7.02	1.75E−11	2	221059443	OpenSea		
cg06384533	−0.63	0.09	−6.97	2.05E−11	6	31789846	OpenSea		
cg11468363	−0.94	0.13	−7.03	2.26E−11	4	87857418	S_Shore	AFF1	5’UTR
cg07944381	0.54	0.08	7.03	2.51E−11	6	31696100	Island	DDAH2	Body
cg27519693	0.68	0.10	6.95	2.61E−11	19	46084524	N_Shelf	OPA3	Body
cg18151858	−0.61	0.08	−7.26	2.62E−11	13	21635991	S_Shore	LATS2	TSS1500
cg14809932	0.80	0.11	7.04	2.64E−11	6	29525723	S_Shelf	UBD	Body
cg25404930	−0.51	0.07	−7.03	2.75E−11	17	58212864	Island		
cg00625110	−0.51	0.07	−7.15	2.86E−11	16	53741731	S_Shelf	FTO	Body
cg18965086	0.79	0.11	7.04	3.07E−11	1	109619546	S_Shore	TAF13	TSS1500
cg07962118	−0.41	0.06	−6.92	3.31E−11	19	49631517	S_Shore	PPFIA3	Body
cg17875102	0.55	0.08	6.89	3.38E−11	6	33926023	OpenSea		
cg01827581	0.67	0.09	7.08	3.58E−11	12	123640508	OpenSea		
cg06536150	2.35	0.33	7.03	3.72E−11	1	2458209	Island	PANK4	TSS200
cg24835539	−0.33	0.05	−6.88	3.83E−11	15	79165346	Island	MORF4L1	5’UTR;1stExon
cg27412506	0.47	0.07	7.02	3.84E−11	15	22954633	OpenSea	CYFIP1	Body; TSS1500
cg04569429	−0.57	0.08	−7.00	3.86E−11	17	37024625	N_Shore	LASP1	TSS1500
cg08211722	−0.77	0.11	−7.01	4.08E−11	22	50708924	Island	MAPK11	TSS200
cg23354319	−0.79	0.11	−6.94	4.29E−11	12	6772506	Island	ING4	TSS200
cg10742797	0.56	0.08	6.86	4.77E−11	1	28672949	OpenSea		
cg09295063	−0.50	0.07	−6.82	5.45E−11	11	118436662	OpenSea	C11orf60	5’UTR;1stExon
cg16476975	−0.51	0.07	−6.91	6.39E−11	7	155164995	Island		
cg06612023	−0.48	0.07	−6.83	6.43E−11	6	28603407	S_Shore		
cg13491462	−0.47	0.07	−6.78	6.58E−11	6	1384491	Island		
cg06998238	−0.33	0.05	−6.80	6.71E−11	19	9695323	Island	ZNF121	TSS200
cg05889321	−0.67	0.10	−6.97	6.94E−11	10	97416837	S_Shore	ALDH18A1	TSS1500
cg27443867	2.35	0.34	6.86	7.24E−11	10	43725376	Island	RASGEF1A	5’UTR
cg25466091	−0.54	0.08	−6.84	7.30E−11	16	1823195	Island	NME3; EME2; MRPS34	TSS1500; TSS200
cg22751080	−0.94	0.14	−6.76	7.32E−11	17	45726749	Island	KPNB1	TSS1500
cg25561290	1.42	0.21	6.76	7.38E−11	1	45265852	Island	PLK3	TSS200
cg19612048	−0.70	0.10	−6.76	7.40E−11	15	44580896	Island	CASC4	TSS200
cg04432965	−0.48	0.07	−6.86	7.92E−11	1	109825781	Island	PSRC1	5’UTR;1stExon
cg00272903	−0.69	0.10	−7.07	8.14E−11	16	28300253	N_Shelf		
cg00020052	−0.45	0.07	−6.82	8.81E−11	2	68546899	Island	CNRIP1	1stExon;5’UTR
cg07205857	0.51	0.08	6.73	8.94E−11	16	455057	S_Shelf	DECR2	Body
cg26647332	−1.01	0.15	−6.76	9.12E−11	10	31073467	N_Shore		
cg16915821	1.03	0.15	6.94	9.86E−11	11	12030187	Island	DKK3	TSS200;5’UTR
cg27219276	0.55	0.08	7.07	1.00E−10	7	150077602	S_Shore	ZNF775	5’UTR

SE, standard error; *p*-value (adjusted).

### 3.2 Region-based EWAS analysis (DMR)

The region-based analysis for main effect of RA identified 520 DMRs with nominal *p* < 0.05 (**Supplementary Table S4**); among them, 14 DMRs remained significant after adjusting for multiple testing with FWER < 0.05 (**Table 3**).

**Table 3:** Top 14 DMRs differentially methylated for RA with family-wise error rate (FWER) < 0.05.

Chro	Start	End	Value	Area	Cluster length	*p*-value	FWER	Gene name	Location
chr13	113968561	113968658	−0.82	1.64	3	2.80E−06	0.005	LAMP1	Body
chr16	88312269	88312422	−0.73	1.45	2	8.39E−06	0.005		
chr12	129331490	129331490	−1.40	1.40	2	5.59E−06	0.005		
chr17	4094606	4094694	−0.65	1.30	2	1.68E−05	0.015	ANKFY1	Body
chr19	6670865	6671045	−0.63	1.26	4	1.68E−05	0.015	LIGHT	
chr6	58777304	58778072	−0.48	1.43	8	1.12E−05	0.02		
chr12	133308663	133310221	−0.49	0.99	16	1.12E−05	0.02	ANKLE2	Body
chr21	11098936	11099431	−0.48	0.95	10	1.12E−05	0.02		
chr9	139996983	139997767	−0.58	1.74	8	1.96E−05	0.025	MAN1B1	Body
chr4	104177163	104177163	−1.08	1.08	1	0.000103	0.035		
chr10	134581820	134581856	−0.55	1.10	5	3.91E−05	0.04	INPP5A	Body
chr16	89945524	89945578	−0.53	1.07	4	3.91E−05	0.04	TCF25	Body
chr7	4257050	4257050	−1.05	1.05	4	0.000117	0.045	SDK1	Body
chr11	47417780	47417818	−0.51	1.03	10	5.03E−05	0.05	SLC39A13	Body

Value: Average of the estimated coefficients (usually representing the difference between two groups) in the region.

Area: Absolute value of the sum of the estimated coefficients in the region.

Cluster length: Number of probes in the cluster (region).

Analysis of DMR in the smoking subset alone revealed two DMRs (FWER < 0.05) (**Supplementary Table S5**), one in the gene body of TNXB and one in the DNM1 gene body.

### 3.3 Functional annotation of gene clusters

**Figure 3:**
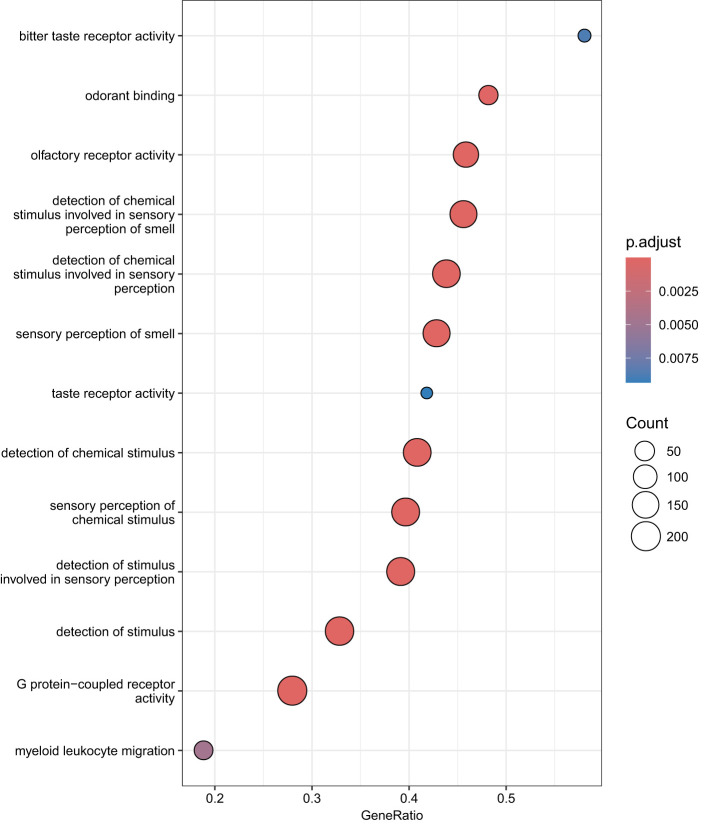
GO enrichment analysis. Dot plots of GSEA results illustrating GO biological processes associated with RA. On the left is the 15 GO terms significantly enriched after correcting for multiple testing. The 15 GO processes with the largest gene ratios are plotted in order of gene ratio. Gene ratio is the fraction of differentially expressed genes found in the gene set. The size of the dots represents the number of genes in the significant methylation gene list associated with the GO term and the color of the dots represent the P-adjusted values.

**Figure 4:**
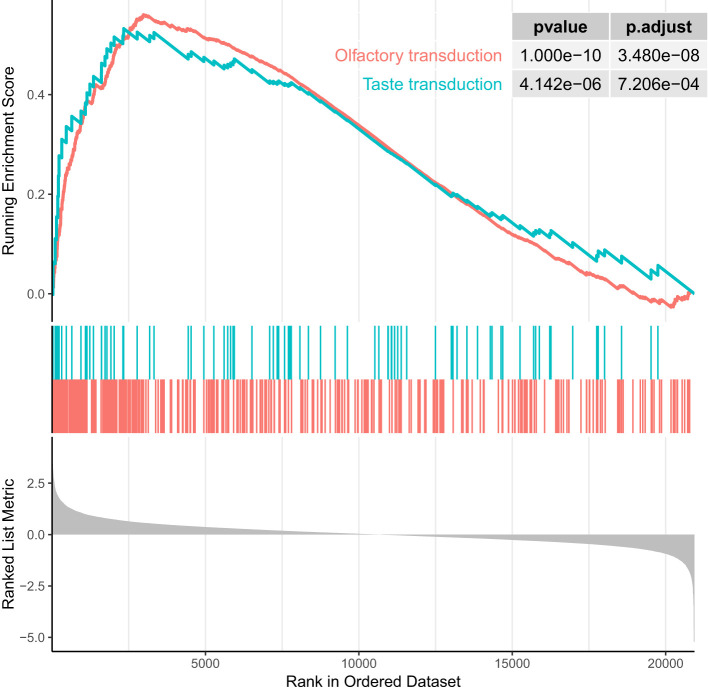
Enrichment plot of top two enrichment KEGG terms (ranked in descending order of normalized enrichment score). P-values are significant (p < 0.001).

We performed GSEA on genes linked to CpG sites associated with RA using GO and KEGG databases. GSEA on GO terms identified 13 GO terms enriched after correcting for multiple testing (**Figure 3**, **Supplementary Table S6**). **Supplementary Figure S2** shows the enrichment curves for the most robust GO terms. GSEA on KEGG pathways only detected two enriched pathways (**Figure 4**), Olfactory transduction with an adjusted *p*-value of 3.33e−08 and Taste transduction with an adjusted *p*-value of 6.07e−04.

ORA of KEGG pathways using the missMethyl method identified four pathways with adjusted *p*-value < 0.1 (**Supplementary Table S7**). The most significant pathway is cell cycle (adjusted *p* of 9.37e−08) followed by viral carcinogenesis (adjusted *p* of 5.22e−04), p53 signaling pathway (adjusted *p* of 7.87e−02), and protein processing in endoplasmic reticulum (adjusted *p* of 9.07e−02).

### 3.4 Replication

A previous EWAS (23), which comprise periferal blood leucocutes (PBL) from treatment-näive ACPA-positive RA patients, identified 51,475 CpGs using the Illumina 450K HumanMethylation Array. When comparing these findings with the present observations, a total of 11,378 out of 16,583 significant CpGs (FDR < 0.05) were identified across the platforms. Among them, 2,248 CpGs overlapped with these authors’ list of significant hits, yielding an overlap rate of 20% (2,248/11,378). A hypergeometric test showed a *p*-value of 2.82e−149, indicating that the agreement of CpGs across these two independent RA populations is highly unlikely to have occurred by chance.

## 4 Discussion

In this exploratory genome-wide study on methylation sites and regions in genomic DNA from peripheral white blood cells in newly diagnosed and DMARD-naïve patients with ACPA-positive RA, we found an excess of hypomethylation in cases versus controls. In total, we identified 16,583 (FDR < 0.05) differentially methylated CpG associated with RA corresponding to 2% of the CpG sites under investigation and 14 DMRs (FWR < 0.05).

It is well known that cis methylation levels are strongly correlated and functionally relevant findings have been generally associated with genomic regions rather than single CpGs. We identified a total of 14 DMRs associated with RA that were all hypomethylated (**Table 3**). The most robustly associated DMR was located in the gene body of *LAMP1* (lysosomal-associated membrane protein 1). *LAMP1* is distributed among autophagic and endolysosomal organelles and is routinely used as a lysosome marker (24). It has been shown to have an important role in lysosomal recruitment in naïve CD4+CD45 RA T cells (25). T cells from RA patients have deficient N-myristoyltransferase (NMT) function, which leads to impairment of lysosomal recruitment of energy sensor AMP-activated protein kinase (*AMPK*). *AMPK* opposes the mTOR Complex 1 (*mTORC1*) signaling pathway via multiple mechanisms (25). Of interest, we found that three CPGs within the TSS200 and one in the body of the *MTOR* gene were hypomethylated in RA (**Supplementary Table S1**). Activation of the *mTORC1* gene is implicated in the inflammatory process in RA, and inhibition of *mTORC1* seems to reduce joint inflammation in RA and to protect against local bone erosions and cartilage loss (26, 27). Of note, we did not find enrichment of the mTOR signaling pathway in the KEGG database.

Another noteworthy DMR was harbored on chromosome 17 in the gene body of *ANKFY1*, which is a Rab (Ras-associated binding protein) that localizes to early endosomes and stimulates their fusion activity. Knockdown of this gene inhibits autophagosome formation (28) and depletion of *YNKFY1* leads to increased autophagosomal number (29). There is evidence to suggest that deregulation of autophagic pathways is implicated in the pathogenesis of RA and that autophagy plays a key role in bone tissue degradation (30). Moreover, antigen-presenting cells need autophagy to perform citrullinated protein presentation.

Another important DMR was located downstream of the *TNFSF14* gene, also known as *LIGHT* (homologous to lymphotoxins), which exhibits inducible expression and competes with HSV glycoprotein D for binding to herpes virus entry mediator (HVEM), a receptor expressed on T lymphocytes. The protein encoded by this gene is a member of the tumor necrosis factor (TNF) ligand family and a ligand for the receptor *TNFRSF14*, which is a member of the TNF receptor superfamily (31). Both *TNFSF14* and *TNFRSF14* have been demonstrated in macrophages from RA synovial tissue. Moreover, *LIGHT* induces expression of metalloproteinase-9 and proinflammatory cytokines like TNF-α and interleukin-6 and IL-8 in macrophages (32). Furthermore, it seems likely that *LIGHT* promotes both *RANKL*-mediated and *RANKL*-independent osteoclast formation in RA and may play a role in both localized and systemic bone loss in RA (33). Both LIGHT mRNA and LIGHT protein have been detected in RA synovial fluid samples and at much higher levels than in synovial fluid from osteoarthritis, and CD4 T cells seem to be a major source of LIGHT in the joints. Stimulation of synovial fibroblasts with recombinant LIGHT upregulates MMP-9 expression, increases surface expression of adhesion molecule CD54, and increases release of IL-6 (34). Both *HVEM* and lymphotoxin β receptor (*LTβr*) [TNF receptor superfamily member 3 (*TNFRSF3*)] have been detected in RA-FLS and LIGHT induces expression of monocyte chemoattractant (33, 35) molecule-1 (MCP-1), interleukin-8, macrophage inflammatory protein-1α, and intercellular adhesion molecule-1 (ICAM-1) via the *LTβr* (36).

The increased concentration of LIGHT in patients with RA raises the possibility that LIGHT may play a role in immunopathogenetic conditions that are associated with localized or systemic bone loss (33, 35). Thus, *LIGHT* is a cytokine involved in the proliferation and activation of RA fibroblast-like synoviocytes and in both cartilage and bone destruction (37).

Multiple RA-associated DMRs belong to non-coding regions. In this context, it should be acknowledged that 90% of causal genetic variants of autoimmune diseases are non-coding with 60% mapping to immune-cell enhancers (38).

Among the most robust DMPs (**Table 2**), we identified *Malat1* (Lung Adenocarcinoma Transcript 1), a highly conserved nuclear retained lncRNA regulating genes at both the transcriptional and post-transcriptional levels. It seems to play an important role in numerous diseases including cancer and inflammation (39). Increased expression of *MALAT1* has been observed in PBMCs from RA patients and predicted clinical outcomes (40). In addition, genetic polymorphisms within *MALAT1* have been associated with genetic susceptibility to RA (41).

*MALAT1* also interacts with and influences the distribution of splicing factors in nuclear speckle domains (42), but was not included as part of the hypothesis testing of impaired splicing machinery identified by a gene expression study of RA patients (43). However, we looked up CpGs from our work that would be in proximity to the 22 differentially expressed genes of splicing in either monocytes, lymphocytes, or neutrophils in their study (43). We studied 31 of their differentially expressed genes. Only 16 of them were differentially methylated and often in the opposite direction. The *RNU4ATAC* gene had the highest expression fold change in monocytes (log10 = 3) and in the neutrophils (log10 = 4), which we found strongly hypomethylated (−435) at the transcription site of peripheral blood white cells, while most of the sites under study were not in accordance with respect to change in methylation and expression. Such a comparison requires careful considerations. Thus, the present study comprised patients with untreated newly diagnosed ACPA-positive disease, while most previous studies included patients with established and treated disease, as well as ACPA-negative cases. Furthermore, the comparisons could also be compromised by differences in power as the expression study had an unfavorable proportion of cases versus controls (129/29) as compared to our study (101/200). Nonetheless, we observed differentially methylated positions in 16 genes potentially involved in the splicing machinery.

Conversely, we found that the 1st Exon of *TWIST1* was hypermethylated. In this region, Liu et al. have previously identified four hypermethylated DMPs associated with RA (23). *TWIST1* is an antagonist of nuclear factor κβ (NF-κβ)-dependent cytokine expression (44). It is expressed in high levels in Th1 effector memory cells in inflamed joints and limits the expression of interferon-γ, IL-2, and TNF-α and ameliorates Th1-mediated immunopathology in antigen-induced arthritis (44). *TWIST1* knockout leads to chronic joint inflammation in a murine arthritis model. Thus, control of inflammation seems to be associated with the *TWIST1* gene and its expression, which is induced by IL-12 via STAT4 and TCR signaling. The proximal promotor of *TWIST1* contains phylogenetically conserved binding sites for nuclear factor-activated T cells (NFAT) and NF-κβ and requires the concerted action by signal transducer and activator of transcription 4 (STAT4). Liu et al. also found additional hypomethylation of the *TWIST2* gene and both proteins seem to be implicated in the regulation of TNF-α production by anti-inflammatory factors and pathways provide a mechanism by which type I interferons and AXL receptor tyrosine kinase suppress inflammatory cytokine production.

We identified two KEGG enriched pathways, bringing attention to less appreciated molecules and pathways as important contributors of RA autoimmunity, namely, the gustatory transduction pathway and the olfactory pathway. In a study by Steinbach et al., both gustatory and olfactory functions were assessed in 101 RA patients with established and treated RA (45). The RA patients had decreased gustatory and olfactory scores compared to the control group. It was speculated if this could be related to systemic inflammation and/or the influence of therapeutic agents, which may induce neuropathies. An effect of therapeutic agents hardly applies to our DMARD-naïve study population. In another study, the volume of the olfactory bulb was reduced in RA patients. Humans have more than 400 smell receptors, G-protein-coupled receptors (GPCRs), but these are not unique to the nose and are expressed in various non-nasal tissues, e.g., kidney, lung, and arteries, of which some are reported to drive atherosclerosis and hypertension (46). Many of the human smell receptors can be expressed by macrophages and cause them to release an inflammatory messenger known to accelerate atherosclerosis. In a genome-wide SNP-based analysis of patients with extreme total carotid plaque involvement, gene sets were significantly enriched in both the KEGG taste transduction pathway and the GO-term “sensory perception of bitter taste” (47). Furthermore, 5 of the top 10 independent SNPs in that study were differentially methylated for the main effect of RA in our study. Of note, RA patients have high-risk carotid plaque generation (47) and the presence of carotid plaques is a predictor of future cardiovascular events and death in patients with RA (48).

According to Geeleher et al. (49), genes with larger numbers of probes are more likely to have significantly differentially methylated CpGs, a situation that can bias pathway-based analysis. The impact of varying numbers of CpGs per gene is also shown by **Supplementary Figure S3** plotting the number of CpGs per gene against the proportion of differential methylation (FDR < 0.05) in our data. With this consideration, we performed additional KEGG pathway analysis using the missMethyl package, which takes into account the number of CpGs per gene in the analysis. Although the method is an over-representation approach instead of an enrichment analysis, some identified pathways (**Supplementary Table S7**) may be functionally meaningful. Thus, RA is characterized by synovial lining hyperplasia, and experimental data suggest that alterations in the expression of proteins involved in maintaining homeostatic control of the cell cycle are involved in disease progression in RA (50, 51). The P53 protein is expressed in RA FLSs, and its overexpression is a characteristic feature of RA (52). Furthermore, endoplasmic reticulum (ER) stress-associated gene signatures are highly expressed in RA synovium and synovial cells and characterized by overexpression of ER stress proteins (53, 54). To our knowledge, the viral carcinogenesis pathway has not previously been associated with RA, although there is much evidence to suggest an association between the pathogenesis of RA and virus, particularly the Epstein–Barr virus (55), and also an increase in Epstein–Barr virus-associated lymphomas in RA (56). Of note, we have previously demonstrated that EBNA1 antibody levels are distinctively increased in healthy, but strongly RA predisposed subjects (57).

We also identified two DMRs, *TNXB* and *DNM1*, that were associated with smoking in RA, one in the gene body of *TNXB* where mutations may predispose to RA through defects in fibrillar collagen structure (57). The second DMR was in the gene body of *DNM1* on chromosome 9. *DNM1* mutations have been associated with severe childhood epilepsy but, to our knowledge, not previously to RA.

The strongest main effect of smoking on RA was associated with hypomethylation of the cg05575921 site in the aryl hydrocarbon receptor repressor (*AHRR*) gene. This site has previously been shown to have the highest level of detectable changes in DNA methylation in whole blood cells from smokers of all kinds (−24.40% methylation; *p* = 2.54E−182) and predicts future smoking-related mortality and morbidity, possibly including RA.

Our study has some limitations. The results were obtained by analysis of DNA from circulating white blood cells, which comprise different cell types in different proportions and at different stages of differentiation. This implies that the present data should be interpreted meticulously with regard to their role in the RA pathogenesis. In order to meet this highly pertinent concern, we adjusted for differences in cell-type composition in our calculations. In addition, we replicated 2,248 DMPs from a large EWAS on peripheral blood leucocytes (PBLs) from a comparable RA population (23), which yielded an overlap rate at 20% with their CpGs. Both the present study and a previous one consisted of recent onset ACPA-positive, treatment-naïve patients retrieved from ethnically homogeneous background populations, and the results were adjusted for age, sex, and smoking habits in both studies.

Furthermore, it is uncertain whether epigenetic marks in RA target tissues, the synovial membrane in particular, are reflected in WBC epigenetic profiles. Nonetheless, it should be kept in mind that the acutely inflamed RA synovial membrane is heavily infiltrated by T and B cells, monocytes, and, last but not least, neutrophils, which play an important role in RA and also display the highest number of hypomethylated regions of all blood cell types, which accords well with their fully differentiated effector phenotype (58). Notably, it has been demonstrated that epigenetic imprinting of synovial fibroblast-like synoviocytes is associated with differentiation into a particularly aggressive phenotype in RA (59). The recent report that RA flares are preceded by the occurrence of blood-borne preinflammatory mesenchymal cells (PRIME cells) that may migrate to the synovial membrane while cross talking with monocytes and neutrophils (60) should stimulate interest in broad epigenetic blood-based profiling and subsequently fine mapping of relevant cell-type patterns.

It should also be kept in mind that although DNA methylation is the best-studied kind of epigenetic modification, alternative mechanisms like, e.g., histone acetylation and altered chromatin structure may contribute additionally to the RA-associated epigenome. Furthermore, although we have adjusted for differences in cell-type composition, we have not done any technical replication of our results. However, EPIC has been shown to have a high reproducibility and reliability (61) and DMRs often encompass multiple CpGs, thereby rendering them more resistant to spurious findings (19). Finally, it cannot be inferred whether the aberrant methylation patterns are causal to RA development, reactive, or incidentally associated with the disease.

Strengths include the fact that, in accordance with current concepts on the diversity of RA, this study solely included ACPA-positive patients. In addition, both patients and controls were of Scandinavian ancestry, and the patients had clinical disease of short duration and had not been treated with DMARDs or glucocorticoids. The patient:control ratio was 1:2, the RA patients were carefully selected and characterized by few board-certified rheumatology specialists, and the analysis was adjusted for age, sex, and smoking habits.

To conclude, DNA is consistently hypomethylated in both coding and non-coding regions in circulating white blood cells from DMARD-naïve patients with newly diagnosed ACPA-positive RA versus controls. This emphasizes the importance of future research also in non-coding regions in relation to the pathogenesis of RA. Among the strongest 14 DMRs in coding regions, we found the *TNSF14* and the *LAMP1* gene. We have also replicated 2,248 RA-associated CpGs from a comparable EWAS in ACPA-positive incident cases, suggesting that particular attention is paid to these sites and regions in future research initiatives in order to elucidate their pathogenetic role. The olfactory and gustatory pathways likely represent a link between systemic RA immune inflammation and neural networks. Finally, our results may contribute to the development of biologically plausible biomarkers to be used in humans at risk of developing RA and to pave the way for emerging and narrowly targeted drugs for the treatment of RA.

## Data Availability

The datasets presented in this article are not readily available because, according to Danish legislation, the transfer and sharing of individual-level data requires prior approval from the Danish Data Protection Agency and requires that the data sharing requests be dealt with on a case-by-case basis: https://www.sdu.dk/en/forskning/dtr/researcher/guidelines#how-to-apply-for-data. Requests to access the datasets should be directed to The Danish Twin Registry, and we encourage you to send a query to tvilling@health.sdu.dk.
