# Supplementary figures and images for: Differential DNA methylation patterns in whole blood from ACPA-positive patients with DMARD-naïve rheumatoid arthritis at clinical disease onset
Source: Front Immunol. 2025 Jul 21;16:1488161. doi: 10.3389/fimmu.2025.1488161 (PMC12318994; doi:10.3389/fimmu.2025.1488161)

# Beta MDS

## 1000 most variable positions

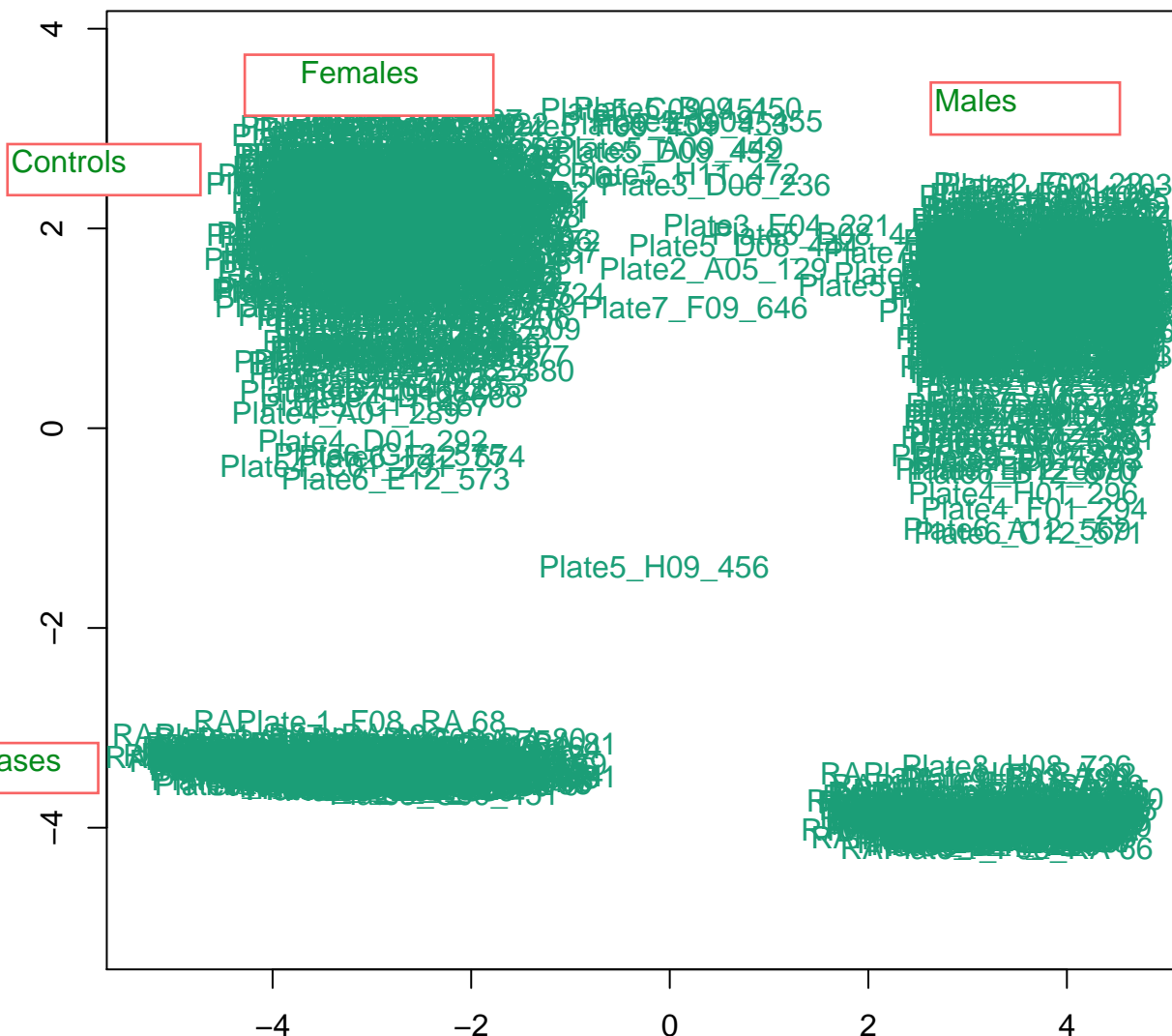

Supplement: Supplementary file 1 [file Image1.pdf]

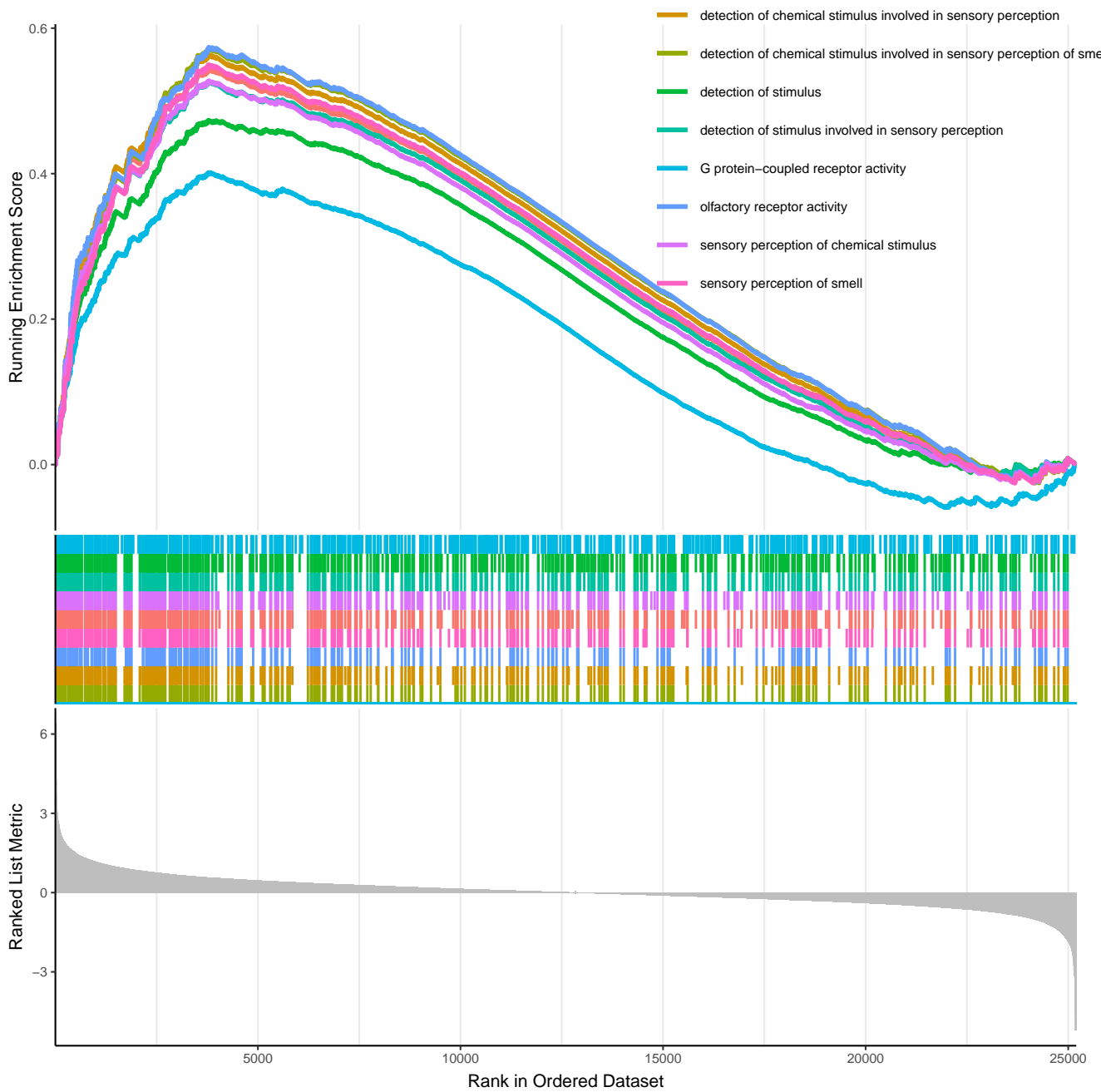

Supplement: Supplementary file 2 [file Image2.pdf]

Proportion Differential Methylation

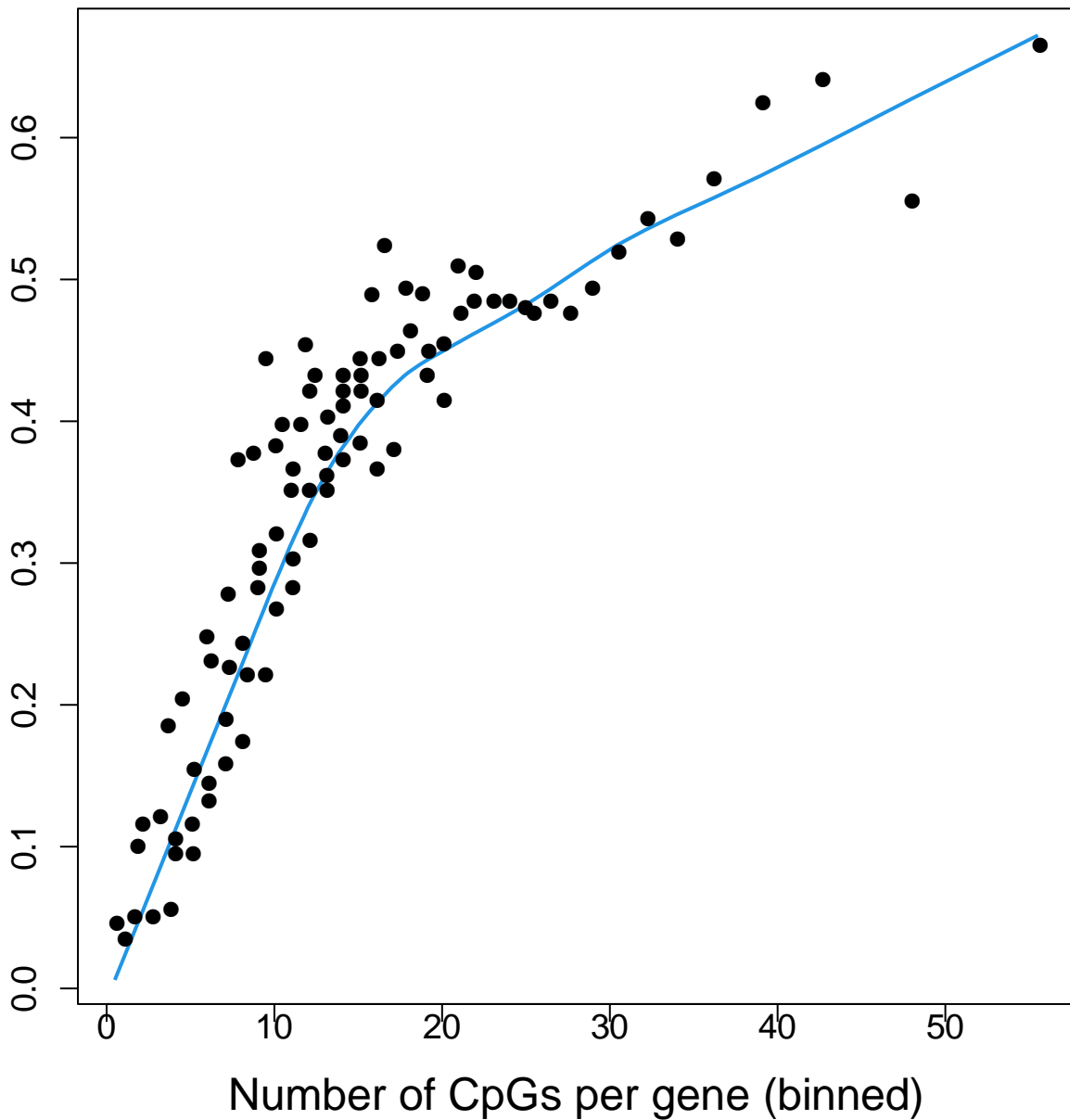

Supplement: Supplementary file 3 [file Image3.pdf]
