# Supplementary material for: Differential DNA methylation patterns in whole blood from ACPA-positive patients with DMARD-naïve rheumatoid arthritis at clinical disease onset
Source: Front Immunol. 2025 Jul 21;16:1488161. doi: 10.3389/fimmu.2025.1488161 (PMC12318994; doi:10.3389/fimmu.2025.1488161)
Supplement: Supplementary file 5 [file Table2.docx]

Supplementary Table 2. General trend of hyper or hypo-methylation by location for significant CpGs with FDR<0.05

|  | All | Promoter* | Gene body* | Intergenic region* |
| --- | --- | --- | --- | --- |
| Number of significant CpGs | 16583 | 2647 | 829 | 578 |
| Hypermethylated | 7339 | 1006 | 376 | 247 |
| Enrichment p | 1 | 1 | 1 | 1 |
| Hypomethylated | 9244 | 1641 | 453 | 331 |
| Enrichment p | 1.45e-130 | 5.25e-112 | 4.97e-7 | 4.32e-10 |

* Exclusive, i.e. CpGs annotated to multiple gene regions are excluded.
