# Supplementary material for: Differential DNA methylation patterns in whole blood from ACPA-positive patients with DMARD-naïve rheumatoid arthritis at clinical disease onset
Source: Front Immunol. 2025 Jul 21;16:1488161. doi: 10.3389/fimmu.2025.1488161 (PMC12318994; doi:10.3389/fimmu.2025.1488161)
Supplement: Supplementary file 11 [file Supplementaryfile1.docx]

*DNA methylation analysis*

Genomic DNA (0.2–1.0 μg) was bisulfite converted using an EZ DNA Methylation-Direct Kit (Zymo Research). DNA was bisulfite converted by incubation with the CT conversion reagent for 8 min at 98 °C, 3.5 h at 64 °C, followed by 18 h at 4 °C in a thermocycler. The treated DNA was added to a Zymo-Spin IC Column, desulfonated using M-desulphonation buffer, and then eluted from the column in 12 μl fractions of M-elution buffer.

Methylation profiling of the bisulfite-treated DNA was performed using Illumina Infinium MethylationEPIC BeadChip (Illumina) according to current standards. In brief, 4 μl of bisulfite-treated DNA was denatured, neutralized and amplified with an overnight whole-genome amplification reaction. The amplified DNA was then enzymatically fragmented, precipitated and re-suspended in hybridization buffer before being dispensed onto the MethylationEPIC BeadChips for hybridization. After hybridization, the BeadChips were processed through a primer-extension protocol and subsequently stained. Finally, the BeadChips were coated and imaged using Illumina’s HiScan System.

*DNA methylation data pre-processing*

The Illumina Infinium MethylationEPIC BeadChip covers 865,859 CpG sites (regions of DNA where a cytosine nucleotide is followed by a guanine nucleotide in the linear sequence of bases along its 5' → 3' direction) across the genome of each individual. Data pre-processing was performed using R package minfi (1) for quality control (QC) and normalization. For probe level QC, a detection p-value was first calculated for each CpG in each sample as 1 − p-value computed from the background model characterizing the chance that the signal was distinguishable from negative controls. A total of 12,227 CpGs with detection p-value > 0.01 in more than 5% of the overall samples were filtered out. A total of 853,632 CpG sites survived probe level QC. Next, we removed all CpGs on the sex chromosomes, filtered out cross-reactive/variant probes using the list provided by Pidsley et al., dropped probes overlapping with SNPs based on columns Probe_maf (SNP in probe within a distance of 50bp), CpG_maf (SNP at CpG), and SBE_maf (SNP at single base extension) from the annotation file, resulting in 687,718 CpGs for subsequent analysis. (2) We further performed sample level QC by plotting the log median intensity of the methylated (M) against the log median intensity of the unmethylated (U) channels using getQC and plotQC functions of minfi, with all samples passed QC. The functional normalization(3) implemented in the minfi R-package was applied for data normalization. Finally, the DNA methylation level at each CpG site was summarized by calculating a methylation “beta” value defined by the Illumina’s formula as β=M/(M+U+100). For the purpose of statistical analysis, the β-values were converted to methylation M-values using logit transformation as M=log2(β/(1- β)).(4)

*Estimating cell-type composition*

As the target tissue in this study is whole blood, cell type heterogeneity is an important factor to be considered due to potential cell type specific DNA methylation. In order to adjust for cell-type composition in the data analysis, the proportions of major white blood cell subsets leukocytes were estimated using the Houseman’s method(5) implemented in the R package minfi. The method used DNA methylation data to estimate blood cell proportions in an individual for 6 blood cell types: CD8T cells, CD4T cells, natural killer cells, B cells, monocytes, and granulocytes. We included the estimated cell type proportions in the association testing to control for the effect of blood cell composition.

Reference List

1. Aryee MJ, Jaffe AE, Corrada-Bravo H, Ladd-Acosta C, Feinberg AP, Hansen KD, et al. Minfi: a flexible and comprehensive Bioconductor package for the analysis of Infinium DNA methylation microarrays. Bioinformatics. 2014;30(10):1363-9.

2. Pidsley R, Zotenko E, Peters TJ, Lawrence MG, Risbridger GP, Molloy P, et al. Critical evaluation of the Illumina MethylationEPIC BeadChip microarray for whole-genome DNA methylation profiling. Genome Biol. 2016;17(1):208.

3. Wu T, Hu E, Xu S, Chen M, Guo P, Dai Z, et al. clusterProfiler 4.0: A universal enrichment tool for interpreting omics data. Innovation (Camb). 2021;2(3):100141.

4. Du P, Zhang X, Huang CC, Jafari N, Kibbe WA, Hou L, et al. Comparison of Beta-value and M-value methods for quantifying methylation levels by microarray analysis. BMC Bioinformatics. 2010;11:587.

5. Houseman E, Accomando W, Koestler D, Christensen B, Marsit C, Nelson H, et al. DNA methylation arrays as surrogate measures of cell mixture distribution. BMC Bioinformatics. 2012;13(1):86.
